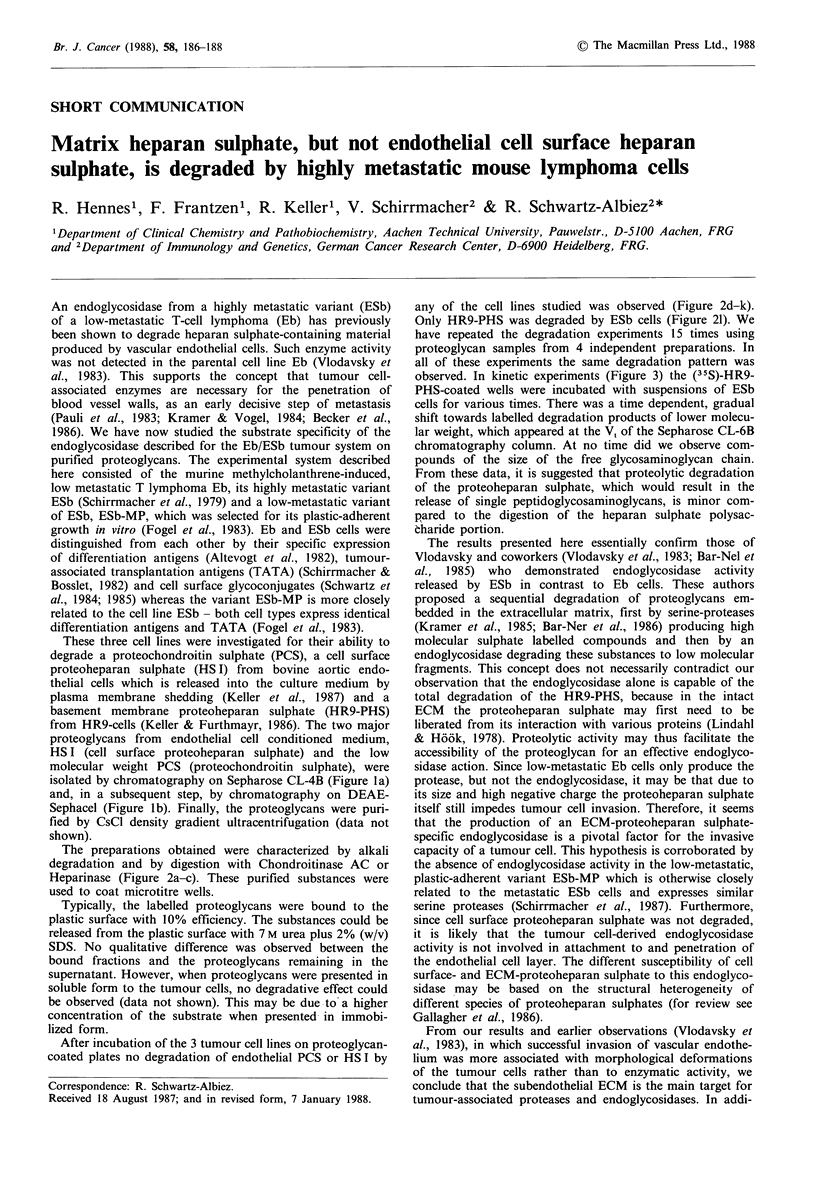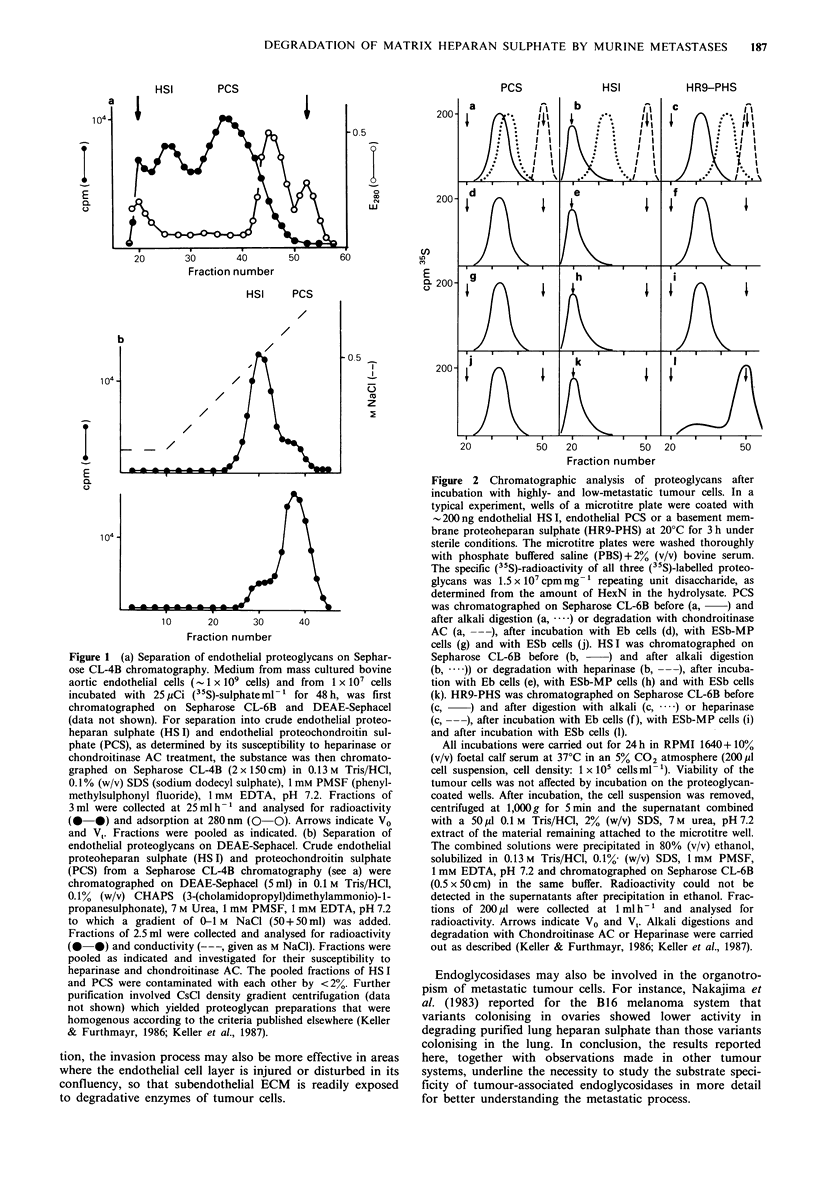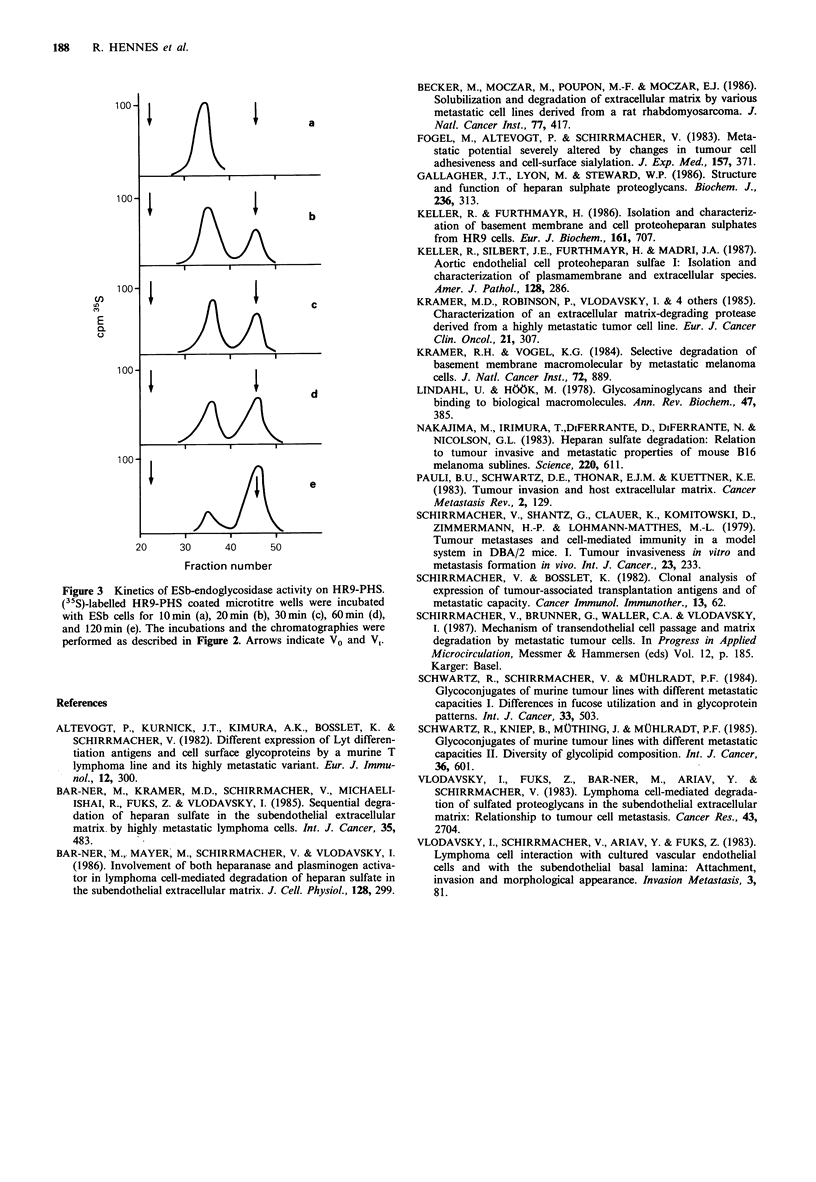# Matrix heparan sulphate, but not endothelial cell surface heparan sulphate, is degraded by highly metastatic mouse lymphoma cells.

**DOI:** 10.1038/bjc.1988.189

**Published:** 1988-08

**Authors:** R. Hennes, F. Frantzen, R. Keller, V. Schirrmacher, R. Schwartz-Albiez

**Affiliations:** Department of Clinical Chemistry and Pathobiochemistry, Aachen Technical University, FRG.


*Br. J. Cancer* (1988), **58**, 186–188 © The Macmillan Press Ltd., 1988

SHORT COMMUNICATION

### Matrix heparan sulphate, but not endothelial cell surface heparan sulphate, is degraded by highly metastatic mouse lymphoma cells

R. Hennes[1], F. Frantzen[1], R. Keller[1], V. Schirrmacher[2] & R. Schwartz-Albiez[2]*

[1]*Department of Clinical Chemistry and Pathobiochemistry, Aachen Technical University, Pauwelstr., D-5100 Aachen, FRG and* [2]*Department of Immunology and Genetics, German Cancer Research Center, D-6900 Heidelberg, FRG.*

An endoglycosidase from a highly metastatic variant (ESb) of a low-metastatic T-cell lymphoma (Eb) has previously been shown to degrade heparan sulphate-containing material produced by vascular endothelial cells. Such enzyme activity was not detected in the parental cell line Eb (Vlodavsky *et al.*, 1983). This supports the concept that tumour cell-associated enzymes are necessary for the penetration of blood vessel walls, as an early decisive step of metastasis (Pauli *et al.*, 1983; Kramer & Vogel, 1984; Becker *et al.*, 1986). We have now studied the substrate specificity of the endoglycosidase described for the Eb/ESb tumour system on purified proteoglycans. The experimental system described here consisted of the murine methylcholanthrene-induced, low metastatic T lymphoma Eb, its highly metastatic variant ESb (Schirrmacher *et al.*, 1979) and a low-metastatic variant of ESb, ESb-MP, which was selected for its plastic-adherent growth *in vitro* (Fogel *et al.*, 1983). Eb and ESb cells were distinguished from each other by their specific expression of differentiation antigens (Altevogt *et al.*, 1982), tumour-associated transplantation antigens (TATA) (Schirrmacher & Bosslet, 1982) and cell surface glycoconjugates (Schwartz *et al.*, 1984; 1985) whereas the variant ESb-MP is more closely related to the cell line ESb – both cell types express identical differentiation antigens and TATA (Fogel *et al.*, 1983).

These three cell lines were investigated for their ability to degrade a proteochondroitin sulphate (PCS), a cell surface proteoheparan sulphate (HS I) from bovine aortic endothelial cells which is released into the culture medium by plasma membrane shedding (Keller *et al.*, 1987) and a basement membrane proteoheparan sulphate (HR9-PHS) from HR9-cells (Keller & Furthmayr, 1986). The two major proteoglycans from endothelial cell conditioned medium, HS I (cell surface proteoheparan sulphate) and the low molecular weight PCS (proteochondroitin sulphate), were isolated by chromatography on Sepharose CL-4B (Figure 1a) and, in a subsequent step, by chromatography on DEAE-Sephacel (Figure 1b). Finally, the proteoglycans were purified by CsCl density gradient ultracentrifugation (data not shown).

The preparations obtained were characterized by alkali degradation and by digestion with Chondroitinase AC or Heparinase (Figure 2a–c). These purified substances were used to coat microtitre wells.

Typically, the labelled proteoglycans were bound to the plastic surface with 10% efficiency. The substances could be released from the plastic surface with 7 M urea plus 2% (w/v) SDS. No qualitative difference was observed between the bound fractions and the proteoglycans remaining in the supernatant. However, when proteoglycans were presented in soluble form to the tumour cells, no degradative effect could be observed (data not shown). This may be due to a higher concentration of the substrate when presented in immobilized form.

After incubation of the 3 tumour cell lines on proteoglycan-coated plates no degradation of endothelial PCS or HS I by any of the cell lines studied was observed (Figure 2d–k). Only HR9-PHS was degraded by ESb cells (Figure 2l). We have repeated the degradation experiments 15 times using proteoglycan samples from 4 independent preparations. In all of these experiments the same degradation pattern was observed. In kinetic experiments (Figure 3) the ($^{35}$S)-HR9-PHS-coated wells were incubated with suspensions of ESb cells for various times. There was a time dependent, gradual shift towards labelled degradation products of lower molecular weight, which appeared at the $V_t$ of the Sepharose CL-6B chromatography column. At no time did we observe compounds of the size of the free glycosaminoglycan chain. From these data, it is suggested that proteolytic degradation of the proteoheparan sulphate, which would result in the release of single peptidoglycosaminoglycans, is minor compared to the digestion of the heparan sulphate polysaccharide portion.

The results presented here essentially confirm those of Vlodavsky and coworkers (Vlodavsky *et al.*, 1983; Bar-Nel *et al.*, 1985) who demonstrated endoglycosidase activity released by ESb in contrast to Eb cells. These authors proposed a sequential degradation of proteoglycans embedded in the extracellular matrix, first by serine-proteases (Kramer *et al.*, 1985; Bar-Ner *et al.*, 1986) producing high molecular sulphate labelled compounds and then by an endoglycosidase degrading these substances to low molecular fragments. This concept does not necessarily contradict our observation that the endoglycosidase alone is capable of the total degradation of the HR9-PHS, because in the intact ECM the proteoheparan sulphate may first need to be liberated from its interaction with various proteins (Lindahl & Höök, 1978). Proteolytic activity may thus facilitate the accessibility of the proteoglycan for an effective endoglycosidase action. Since low-metastatic Eb cells only produce the protease, but not the endoglycosidase, it may be that due to its size and high negative charge the proteoheparan sulphate itself still impedes tumour cell invasion. Therefore, it seems that the production of an ECM-proteoheparan sulphate-specific endoglycosidase is a pivotal factor for the invasive capacity of a tumour cell. This hypothesis is corroborated by the absence of endoglycosidase activity in the low-metastatic, plastic-adherent variant ESb-MP which is otherwise closely related to the metastatic ESb cells and expresses similar serine proteases (Schirrmacher *et al.*, 1987). Furthermore, since cell surface proteoheparan sulphate was not degraded, it is likely that the tumour cell-derived endoglycosidase activity is not involved in attachment to and penetration of the endothelial cell layer. The different susceptibility of cell surface- and ECM-proteoheparan sulphate to this endoglycosidase may be based on the structural heterogeneity of different species of proteoheparan sulphates (for review see Gallagher *et al.*, 1986).

From our results and earlier observations (Vlodavsky *et al.*, 1983), in which successful invasion of vascular endothelium was more associated with morphological deformations of the tumour cells rather than to enzymatic activity, we conclude that the subendothelial ECM is the main target for tumour-associated proteases and endoglycosidases. In addi-

Correspondence: R. Schwartz-Albiez.
Received 18 August 1987; and in revised form, 7 January 1988.

**Figure 1** (a) Separation of endothelial proteoglycans on Sepharose CL-4B chromatography. Medium from mass cultured bovine aortic endothelial cells (~$1 \times 10^9$ cells) and from $1 \times 10^7$ cells incubated with 25 μCi ($^{35}$S)-sulphate ml$^{-1}$ for 48 h, was first chromatographed on Sepharose CL-6B and DEAE-Sephacel (data not shown). For separation into crude endothelial proteoheparan sulphate (HS I) and endothelial proteochondroitin sulphate (PCS), as determined by its susceptibility to heparinase or chondroitinase AC treatment, the substance was then chromatographed on Sepharose CL-4B ($2 \times 150$ cm) in 0.13 M Tris/HCl, 0.1% (w/v) SDS (sodium dodecyl sulphate), 1 mM PMSF (phenylmethylsulphonyl fluoride), 1 mM EDTA, pH 7.2. Fractions of 3 ml were collected at 25 ml h$^{-1}$ and analysed for radioactivity (●—●) and adsorption at 280 nm (○—○). Arrows indicate $V_0$ and $V_t$. Fractions were pooled as indicated. (b) Separation of endothelial proteoglycans on DEAE-Sephacel. Crude endothelial proteoheparan sulphate (HS I) and proteochondroitin sulphate (PCS) from a Sepharose CL-4B chromatography (see a) were chromatographed on DEAE-Sephacel (5 ml) in 0.1 M Tris/HCl, 0.1% (w/v) CHAPS (3-(cholamidopropyl)dimethylammonio)-1-propanesulphonate), 7 M Urea, 1 mM PMSF, 1 mM EDTA, pH 7.2 to which a gradient of 0–1 M NaCl (50+50 ml) was added. Fractions of 2.5 ml were collected and analysed for radioactivity (●—●) and conductivity (– – –, given as M NaCl). Fractions were pooled as indicated and investigated for their susceptibility to heparinase and chondroitinase AC. The pooled fractions of HS I and PCS were contaminated with each other by $< 2\%$. Further purification involved CsCl density gradient centrifugation (data not shown) which yielded proteoglycan preparations that were homogenous according to the criteria published elsewhere (Keller & Furthmayr, 1986; Keller *et al.*, 1987).

tion, the invasion process may also be more effective in areas where the endothelial cell layer is injured or disturbed in its confluency, so that subendothelial ECM is readily exposed to degradative enzymes of tumour cells.

**Figure 2** Chromatographic analysis of proteoglycans after incubation with highly- and low-metastatic tumour cells. In a typical experiment, wells of a microtitre plate were coated with ~200 ng endothelial HS I, endothelial PCS or a basement membrane proteoheparan sulphate (HR9-PHS) at 20°C for 3 h under sterile conditions. The microtitre plates were washed thoroughly with phosphate buffered saline (PBS)+2% (v/v) bovine serum. The specific ($^{35}$S)-radioactivity of all three ($^{35}$S)-labelled proteoglycans was $1.5 \times 10^7$ cpm mg$^{-1}$ repeating unit disaccharide, as determined from the amount of HexN in the hydrolysate. PCS was chromatographed on Sepharose CL-6B before (a, ——) and after alkali digestion (a, ····) or degradation with chondroitinase AC (a, – – –), after incubation with Eb cells (d), with ESb-MP cells (g) and with ESb cells (j). HS I was chromatographed on Sepharose CL-6B before (b, ——) and after alkali digestion (b, ····)) or degradation with heparinase (b, – – –), after incubation with Eb cells (e), with ESb-MP cells (h) and with ESb cells (k). HR9-PHS was chromatographed on Sepharose CL-6B before (c, ——) and after digestion with alkali (c, ····) or heparinase (c, – – –), after incubation with Eb cells (f), with ESb-MP cells (i) and after incubation with ESb cells (l).

All incubations were carried out for 24 h in RPMI 1640+10% (v/v) foetal calf serum at 37°C in an 5% $CO_2$ atmosphere (200 μl cell suspension, cell density: $1 \times 10^5$ cells ml$^{-1}$). Viability of the tumour cells was not affected by incubation on the proteoglycan-coated wells. After incubation, the cell suspension was removed, centrifuged at 1,000 *g* for 5 min and the supernatant combined with a 50 μl 0.1 M Tris/HCl, 2% (w/v) SDS, 7 M urea, pH 7.2 extract of the material remaining attached to the microtitre well. The combined solutions were precipitated in 80% (v/v) ethanol, solubilized in 0.13 M Tris/HCl, 0.1%· (w/v) SDS, 1 mM PMSF, 1 mM EDTA, pH 7.2 and chromatographed on Sepharose CL-6B ($0.5 \times 50$ cm) in the same buffer. Radioactivity could not be detected in the supernatants after precipitation in ethanol. Fractions of 200 μl were collected at 1 ml h$^{-1}$ and analysed for radioactivity. Arrows indicate $V_0$ and $V_t$. Alkali digestions and degradation with Chondroitinase AC or Heparinase were carried out as described (Keller & Furthmayr, 1986; Keller *et al.*, 1987).

Endoglycosidases may also be involved in the organotropism of metastatic tumour cells. For instance, Nakajima *et al.* (1983) reported for the B16 melanoma system that variants colonising in ovaries showed lower activity in degrading purified lung heparan sulphate than those variants colonising in the lung. In conclusion, the results reported here, together with observations made in other tumour systems, underline the necessity to study the substrate specificity of tumour-associated endoglycosidases in more detail for better understanding the metastatic process.

**Figure 3** Kinetics of ESb-endoglycosidase activity on HR9-PHS. ($^{35}S$)-labelled HR9-PHS coated microtitre wells were incubated with ESb cells for 10 min (a), 20 min (b), 30 min (c), 60 min (d), and 120 min (e). The incubations and the chromatographies were performed as described in **Figure 2**. Arrows indicate $V_0$ and $V_t$.

### References

ALTEVOGT, P., KURNICK, J.T., KIMURA, A.K., BOSSLET, K. & SCHIRRMACHER, V. (1982). Different expression of Lyt differentiation antigens and cell surface glycoproteins by a murine T lymphoma line and its highly metastatic variant. *Eur. J. Immunol.*, **12**, 300.

BAR-NER, M., KRAMER, M.D., SCHIRRMACHER, V., MICHAELI-ISHAI, R., FUKS, Z. & VLODAVSKY, I. (1985). Sequential degradation of heparan sulfate in the subendothelial extracellular matrix by highly metastatic lymphoma cells. *Int. J. Cancer*, **35**, 483.

BAR-NER, M., MAYER, M., SCHIRRMACHER, V. & VLODAVSKY, I. (1986). Involvement of both heparanase and plasminogen activator in lymphoma cell-mediated degradation of heparan sulfate in the subendothelial extracellular matrix. *J. Cell. Physiol.*, **128**, 299.

BECKER, M., MOCZAR, M., POUPON, M.-F. & MOCZAR, E.J. (1986). Solubilization and degradation of extracellular matrix by various metastatic cell lines derived from a rat rhabdomyosarcoma. *J. Natl. Cancer Inst.*, **77**, 417.

FOGEL, M., ALTEVOGT, P. & SCHIRRMACHER, V. (1983). Metastatic potential severely altered by changes in tumour cell adhesiveness and cell-surface sialylation. *J. Exp. Med.*, **157**, 371.

GALLAGHER, J.T., LYON, M. & STEWARD, W.P. (1986). Structure and function of heparan sulphate proteoglycans. *Biochem. J.*, **236**, 313.

KELLER, R. & FURTHMAYR, H. (1986). Isolation and characterization of basement membrane and cell proteoheparan sulphates from HR9 cells. *Eur. J. Biochem.*, **161**, 707.

KELLER, R., SILBERT, J.E., FURTHMAYR, H. & MADRI, J.A. (1987). Aortic endothelial cell proteoheparan sulfae I: Isolation and characterization of plasmamembrane and extracellular species. *Amer. J. Pathol.*, **128**, 286.

KRAMER, M.D., ROBINSON, P., VLODAVSKY, I. & 4 others (1985). Characterization of an extracellular matrix-degrading protease derived from a highly metastatic tumor cell line. *Eur. J. Cancer Clin. Oncol.*, **21**, 307.

KRAMER, R.H. & VOGEL, K.G. (1984). Selective degradation of basement membrane macromolecular by metastatic melanoma cells. *J. Natl. Cancer Inst.*, **72**, 889.

LINDAHL, U. & HÖÖK, M. (1978). Glycosaminoglycans and their binding to biological macromolecules. *Ann. Rev. Biochem.*, **47**, 385.

NAKAJIMA, M., IRIMURA, T.,DIFERRANTE, D., DIFERRANTE, N. & NICOLSON, G.L. (1983). Heparan sulfate degradation: Relation to tumour invasive and metastatic properties of mouse B16 melanoma sublines. *Science*, **220**, 611.

PAULI, B.U., SCHWARTZ, D.E., THONAR, E.J.M. & KUETTNER, K.E. (1983). Tumour invasion and host extracellular matrix. *Cancer Metastasis Rev.*, **2**, 129.

SCHIRRMACHER, V., SHANTZ, G., CLAUER, K., KOMITOWSKI, D., ZIMMERMANN, H.-P. & LOHMANN-MATTHES, M.-L. (1979). Tumour metastases and cell-mediated immunity in a model system in DBA/2 mice. I. Tumour invasiveness *in vitro* and metastasis formation *in vivo*. *Int. J. Cancer.*, **23**, 233.

SCHIRRMACHER, V. & BOSSLET, K. (1982). Clonal analysis of expression of tumour-associated transplantation antigens and of metastatic capacity. *Cancer Immunol. Immunother.*, **13**, 62.

SCHIRRMACHER, V., BRUNNER, G., WALLER, C.A. & VLODAVSKY, I. (1987). Mechanism of transendothelial cell passage and matrix degradation by metastatic tumour cells. In *Progress in Applied Microcirculation*, Messmer & Hammersen (eds) Vol. 12, p. 185. Karger: Basel.

SCHWARTZ, R., SCHIRRMACHER, V. & MÜHLRADT, P.F. (1984). Glycoconjugates of murine tumour lines with different metastatic capacities I. Differences in fucose utilization and in glycoprotein patterns. *Int. J. Cancer*, **33**, 503.

SCHWARTZ, R., KNIEP, B., MÜTHING, J. & MÜHLRADT, P.F. (1985). Glycoconjugates of murine tumour lines with different metastatic capacities II. Diversity of glycolipid composition. *Int. J. Cancer*, **36**, 601.

VLODAVSKY, I., FUKS, Z., BAR-NER, M., ARIAV, Y. & SCHIRRMACHER, V. (1983). Lymphoma cell-mediated degradation of sulfated proteoglycans in the subendothelial extracellular matrix: Relationship to tumour cell metastasis. *Cancer Res.*, **43**, 2704.

VLODAVSKY, I., SCHIRRMACHER, V., ARIAV, Y. & FUKS, Z. (1983). Lymphoma cell interaction with cultured vascular endothelial cells and with the subendothelial basal lamina: Attachment, invasion and morphological appearance. *Invasion Metastasis*, **3**, 81.